# Comparing the pericapsular nerve group block and fascia iliaca block for acute pain management in patients with hip fracture: a randomised clinical trial

**DOI:** 10.1111/anae.16695

**Published:** 2025-07-29

**Authors:** Santi Di Pietro, Riccardo Maffeis, Eugenio Jannelli, Benedetta Mascia, Flavia Resta, Annalisa De Silvestri, Valeria Musella, Clarissa Elisabeth Centurioni, Elena Regeni, Federico Alberto Grassi, Alessandro Locatelli, Stefano Perlini, the Regional Anaesthesia in Emergency Medicine (RAEM) Research Group, Federica Alini, Valentina Angeli, Bruno Barcella, Rebecca Bersani, Clara Bettini, Marco Bonzano, Vincenzo Capozza, Letizia Caseti, Alberto Castelli, Annalisa Ceruti, Pietro Costa, Carlotta Cremaschi, Fabrizio Cuzzocrea, Salvatore D'Amico, Emanuele Dal Fuoco, Andrea Simone Dedato, Guido Forini, Federica Fossati, Matteo Ghiara, Elena Lago, Irene Macaluso, Luca Mangini, Sara Mariucci, Giuseppe Mignosa, Greta Monne, Mario Mosconi, Cristina Naturale, Fabrizio Paparella, Nicola Parisi, Gianluigi Pasta, Claudio Pavesi, Francesco Pelillo, Michela Alessandra Pierro, Michele Rendina, Francesco Salinaro, Annunziata Santaniello, Valeria Sergi, Antonino Tavella, Damiano Vignaroli, Jessica Zanovello

**Affiliations:** ^1^ Emergency Medicine Research Unit, Department of Internal Medicine and Therapeutics University of Pavia Pavia Italy; ^2^ PhD Program in Experimental Medicine University of Pavia Pavia Italy; ^3^ Emergency Medicine Research Oxford (EMROx) Oxford University Hospitals NHS, Foundation Trust Oxford UK; ^4^ Operative Unit of Orthopaedics and Traumatology, IRCCS Fondazione Policlinico San Matteo, Department of Clinical, Surgical, Diagnostic and Pediatric Sciences University of Pavia Pavia Italy; ^5^ Anaesthesia and Reanimation Service IRCCS Fondazione Policlinico San Matteo Pavia Italy; ^6^ Biostatistics and Clinical Trial Center IRCCS Fondazione Policlinico San Matteo Pavia Italy; ^7^ Emergency Medicine Residency Program Vita‐Salute San Raffaele University Milan Italy; ^8^ Emergency Medicine Residency Program University of Trieste Trieste Italy

**Keywords:** acute pain, analgesia, fascia iliaca block, pericapsular nerve group block, regional anaesthesia

## Abstract

**Introduction:**

The fascia iliaca block (FIB) is currently recommended as a component of multimodal acute pain management for patients with hip fracture. The pericapsular nerve group (PENG) block is a newer technique that may provide superior analgesia. We therefore designed this study in an academic emergency department to compare the acute analgesic effect of these two approaches in patients with a hip fracture.

**Methods:**

Adult patients with an acute hip fracture who reported at least moderate pain were eligible for inclusion. Patients were allocated randomly to receive either a PENG block with 20 ml 0.375% levobupivacaine plus 4 mg dexamethasone or infra‐inguinal FIB with 30 ml 0.25% levobupivacaine plus 4 mg dexamethasone. Primary outcome was the percentage of summed pain intensity difference (%SPID) calculated from visual analogue pain scores measured during the first hour post‐block. Secondary outcomes included: number of patients reaching 33% and 50% SPID; dose of rescue opioid administered in morphine milligram equivalents; and incidence of adverse events.

**Results:**

In total, 92 patients were screened for eligibility and 64 were enrolled (32 in each group). Patients allocated to the PENG block group showed a greater %SPID when compared with those allocated to the FIB group (62.7% (95%CI 52.9–72.4%) vs. 38.0% (95%CI 30.7–45.4%), respectively; difference: ‐24.7% (95%CI ‐36.6 to ‐12.7%), p < 0.001). In the PENG group, 24/32 patients achieved 50% SPID compared with 7/32 in the FIB group (p < 0.001). Similarly, in the PENG group, 28/32 patients achieved 33% SPID compared with 19/32 in the FIB group (p = 0.022). There was no significant difference in the rescue opioid dose administered or in the incidence of adverse events.

**Discussion:**

The PENG block provides superior analgesia for the first hour after intervention when compared with the infra‐inguinal FIB and represents a promising modality for acute pain management in emergency departments.

## Plain Language Summary

When people break their hip, they usually feel a lot of pain. Doctors use special kinds of pain relief to help them feel better. This includes nerve blocks to make the hip feel numb. One type is called the fascia iliaca block, and another newer one is the PENG block. This study was done in a hospital emergency department to see which block worked better at reducing pain quickly for people with a hip fracture. Doctors randomly chose which block each patient would get. They picked patients who had a broken hip and were in moderate or worse pain. One group got the PENG block, using 20 ml of a numbing medicine with a small amount of another helpful drug. The other group got the fascia iliaca block, using a little more of a slightly weaker numbing medicine and the same extra drug. The main goal was to see how much the pain went down after the block. Other things they checked were: how many patients had at least a third or half less pain; how much extra pain medicine (like morphine) was needed; and if anyone had bad side effects. Out of 92 patients checked, 64 joined the study (32 in each group). Patients who got the PENG block had a bigger drop in pain than those who got the fascia iliaca block. In the PENG block group, 28 out of 32 patients felt at least a third less pain, compared to 19 out of 32 in the other group. Both groups needed about the same amount of extra pain medicine. The PENG block helped reduce pain better in the first hour after treatment than the fascia iliaca block. It looks like a good option for helping patients with hip fractures feel better in the emergency department.

## Introduction

Hip fracture is a common injury sustained by older people and is associated with severe acute pain. In the early phases of care, patients with a hip fracture must be mobilised multiple times for transport from the site of injury to hospital, admission to the emergency department, assessment by emergency medicine staff and consultants, and the performance of imaging studies to establish a diagnosis. Hospital admission and definitive surgical treatment may be delayed due to healthcare system issues such as lack of inpatient beds and surgical waiting lists [1]. Therefore, as these patients spend more time in emergency departments, implementing timely and consistent multimodal analgesia before hospital admission is paramount because poorly managed pre‐operative pain contributes to negative clinical outcomes [2, 3].

The use of regional block techniques to provide pre‐operative non‐opioid analgesia in patients with hip fracture has been recommended by medical societies, including the Royal College of Emergency Medicine best practice guideline on the use of fascia iliaca block in the emergency department [4, 5, 6]. The potential advantages of early nerve block interventions include improved analgesia at the site of injury and decreased need for opioids and other sedatives, which is particularly desirable in an older and frailer population who are at risk of delirium [7, 8]. In the peri‐operative period, use of nerve blocks for analgesia may facilitate early mobilisation after surgery and decrease hospitalisation duration, thereby reducing potential hospital‐acquired complications like pneumonia or venous thromboembolism [8].

In many healthcare systems worldwide, anaesthetists are not immediately available 24 h per day, especially for patients in the emergency department. For this reason, basic nerve block techniques have been incorporated into emergency medicine residency curricula, and the fascia iliaca block has been identified by emergency medicine physicians as a preferred intervention in the acute pain management of patients suffering from a hip fracture [4, 9]. Despite supportive evidence [8], the fascia iliaca block may not always be the ideal nerve block option in the setting of enhanced recovery because it consistently produces quadriceps motor block by anaesthetising the femoral nerve and yet does not anaesthetise the hip joint reliably [10].

The pericapsular nerve group (PENG) block is a newer technique designed to target the anterior hip joint by depositing local anaesthetic in a fascial plane containing the terminal sensory fibres from the femoral, obturator and accessory obturator nerves [11]. Several studies conducted in the context of anaesthesia and peri‐operative care have reported the benefits of the PENG block when compared with the fascia iliaca block [12, 13, 14, 15, 16, 17, 18, 19]. A recent meta‐analysis concludes that the PENG block may be superior to the fascia iliaca block for patients with a hip fracture, but these results cannot be considered definitive due to the high risk of bias and heterogeneity among the included studies and questions regarding data integrity [20, 21].

Since determining which block is superior has direct relevance to patients who present to emergency departments with acute pain and current evidence is limited to anaesthetic care, we designed this randomised clinical trial in the emergency department setting to test the hypothesis that the PENG block provides superior acute pain management compared with the fascia iliaca block in patients who present with a hip fracture, as measured within 1 h of block performance.

## Methods

Approval for this study was granted by the regional research ethics committee. All participants provided written informed consent before enrolment. The trial protocol adhered to the SPIRIT statement [22] and the reporting of the trial adhered to CONSORT guideline [23].

This study was initially designed as a dual‐centre international randomised clinical trial involving the emergency departments at the sponsoring institution IRCCS Fondazione Policlinico San Matteo, Pavia, Italy, and Colchester General Hospital, Colchester, United Kingdom (online Supporting Information Appendix S2).

Patients were invited to participate by one of the trained researchers after verifying eligibility and the availability of an emergency medicine consultant trained in both block techniques. We offered study participation to adult patients (≥ 18 y) in the emergency department who had: a radiologically confirmed proximal femur fracture (subcapitate, transcervical, intertrochanteric or pertrochanteric); moderate to severe acute pain (> 40 mm) at rest or with movement on the 100‐mm visual analogue scale (VAS, 0 mm = no pain; 100 mm = worst possible pain); and capacity to provide their own consent and self‐assessment of pain using the written VAS. Patients with known hypersensitivity to local anaesthetics and/or paracetamol; subtrochanteric, diaphyseal or periprosthetic fractures; haemodynamic instability; history of severe cognitive impairment; evidence of dementia or delirium at assessment (defined by a 4AT score ≥ 2) [24]; BMI > 35 kg.m^‐2^; body weight < 40 kg; or worst pain on the VAS ≤ 40 mm at rest or with movement were not studied. A more detailed description of the inclusion and exclusion criteria is included in online Supporting Information Appendix S2.

After signing consent, patients were taught how to use the VAS and were then provided with five sheets of paper with a 100‐mm VAS line on each and asked to report their pain level using the VAS at baseline before the block was performed (T0). Pain was assessed both at rest and with movement (hip flexion to 15° with gentle passive straight leg raise of the affected limb), and the worst pain during this latter dynamic assessment was recorded.

We used the online platform REDCap for randomisation (Vanderbilt University, Nashville, TN, USA) and generated a 1:1 block randomisation list using the ‘ralloc’ procedure in STATA v17.0 (StataCorp, College Station, TX, USA). The recruiting researcher used REDCap to allocate patients 1:1 to receive a PENG or fascia iliaca block. Patients were not informed of their group allocation, and to blind them, the ultrasound screen was turned away from patient view, and the same area of skin in the groin region was disinfected for both block techniques. The operator performing the procedures was not blinded, but all other treating clinicians were not informed of group allocation.

After the baseline pain assessment and before receiving either block, all patients could receive 15 mg/kg^‐1^ of paracetamol intravenously for pain relief if no paracetamol had been received within the previous 6 h. No patients received anxiolytics or opioids for the block procedure itself. All blocks were performed by a small group of senior emergency medicine consultants who were trained in both techniques. Although only four physicians formed this group, two physicians left the institution during the conduct of the study and were replaced by two emergency medicine physicians who were also trained in both block techniques. Therefore, a total of six physicians performed all procedures.

Patients allocated to the PENG block group received an ultrasound‐guided PENG block as described by Girón‐Arango et al. [11]. Briefly, relevant sono‐anatomy was identified at the level of the inguinal ligament using a 2–6 MHz curvilinear transducer (SonoSite Edge ll, Bothell, WA, USA). Using in‐plane guidance, an 18‐gauge, 90‐mm needle (Stimuplex D, B. Braun, Melsungen, Germany) was inserted until the tip contacted the iliopubic eminence under the psoas tendon. After saline injection (up to 2 ml) to confirm needle tip position and hydrodissection of the correct plane, 20 ml 0.375% levobupivacaine plus 4 mg dexamethasone were injected incrementally, with anterior displacement of the psoas tendon confirming successful deposition of the injectate. For patients allocated to the fascia iliaca block group, relevant sono‐anatomy was identified caudal to the inguinal ligament (infra‐inguinal approach) using a 4–16 MHz linear transducer. The block was performed using the same type of needle used for the PENG block procedures, which was guided in‐plane from lateral to medial until the tip penetrated the fascia iliaca at approximately the level of the junction between the middle and lateral thirds of the inguinal crease [4]. Once the needle tip position was confirmed with saline injection, 30 ml 0.25% levobupivacaine plus 4 mg dexamethasone was injected incrementally, with separation of the fascia iliaca from the iliacus muscle and injectate spread medially towards the femoral nerve confirming successful deposition of injectate.

After block completion, the primary treating clinician (blinded to group allocation) assessed pain at 5 min, 15 min, 30 min and 60 min post‐block (T1, T2, T3 and T4, respectively) using the standardised procedure described previously. In total, 17 different emergency department clinicians performed assessments for the entire study. We limited the interval for pain measurements to 1 h post‐block to maximise attribution of any analgesic effect to the intervention, reduce the risk of heterogeneous data resulting from patient movement (e.g. subsequent clinical evaluations, imaging studies, transportation) and avoid overlap with the peri‐operative period. No formal clinical assessment of block success was performed as the PENG block does not reliably result in cutaneous sensory changes [25]. Rescue analgesia was available to all patients in the form of an intravenous bolus of morphine 0.05 mg.kg^‐1^, which could be repeated once after 30 min if needed for unrelieved severe acute pain. The primary outcome was pain relief over the 60‐min interval following block performance, which was measured as the percentage of summed pain intensity difference (%SPID) using VAS pain scores reported at the five time points described previously [26].

Secondary outcomes were: the number (proportion) of patients who achieved 33% and 50% of the maximum SPID, with 33% of the SPID being identified previously as a clinically significant threshold [25]; dose of opioid in intravenous morphine milligram equivalents (MME) administered in the 60 min after block; and incidence of adverse events during the entire emergency department stay (nausea or vomiting, hypotension (systolic blood pressure < 100 mmHg), respiratory depression (< 10 breaths.min^‐1^, oxygen saturation < 94% or naloxone administration) or local anaesthetic systemic toxicity).

Beaudoin et al. described a median (range) %SPID of 36.9 (‐25–100) following the femoral ‘3‐in‐1’ block [26], from which we derived the standard deviation of 33 using the formula described by Wan et al. [27]. The femoral ‘3‐in‐1’ block and fascia iliaca block with ultrasound guidance essentially target the same nerves and have been shown to provide similar levels of analgesia [28]; therefore, we expected similar %SPID in the fascia iliaca block group. At the time of study design, the PENG block was reported to reduce pain by 7 points on an 11‐point pain scale 30 min post‐block, from which we derived a mean %SPID of 65 [29, 30, 31]. To provide 90% power with an α of 0.05, enrolment ratio of 1:1 and a common SD of 30, a sample size of 29 participants in each group was estimated. We increased the sample size by 10% to account for attrition, missing data and possible protocol violations, resulting in a total of 32 participants in each group.

Stata v17.0 (StataCorp LLC, College Station, TX, USA) was used for statistical analyses. Normality of distribution was determined for all continuous variables by the Shapiro–Wilk test. For outcomes with continuous variables, treatment groups were compared using the *t*‐test for independent samples if normally distributed or the Mann–Whitney U‐test if not normally distributed. For comparisons of proportions, Pearson's χ^2^ test was used or Fisher's exact test when appropriate (n < 5 in any field). A p < 0.05 was considered statistically significant. In all cases, two‐tailed tests were applied.

## Results

Between August 2022 and August 2024, 92 patients were screened for eligibility. In order to screen and recruit patients for this trial, an investigator and a physician trained in both block techniques had to be present simultaneously in the emergency department. The relatively small number of trained physicians and opportunistic nature of recruitment (given the inability to predict when patients would present with hip fractures) likely influenced the number of patients screened and recruited. In addition, the planned second recruiting centre in the UK could not be established in time to join the study. In order to submit an amendment to the study protocol, recruitment was interrupted on 26 February 2023, limiting patient enrolment to the single sponsoring institution, and restarted on 3 July 2023 after research ethics committee approval of the amendment.

**Figure 1:**
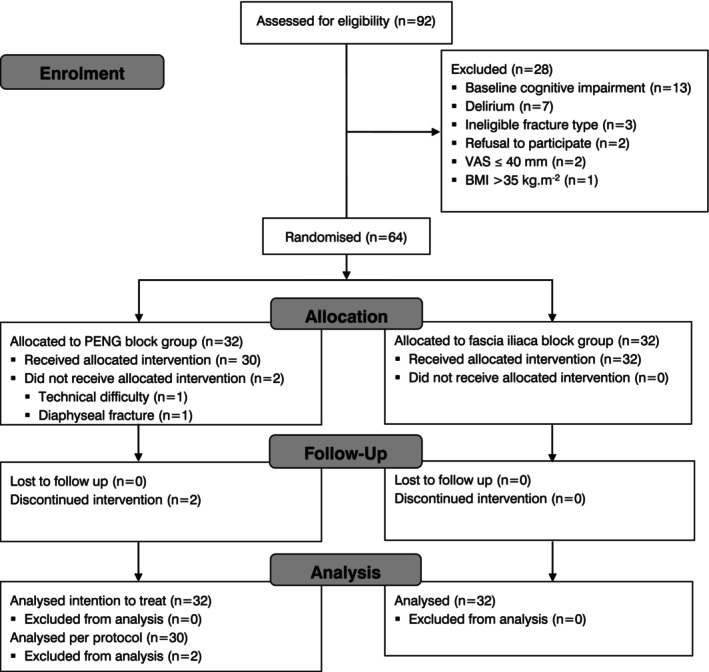
Study flow diagram.

From the 92 patients screened, 28 were not included: baseline cognitive impairment (n = 13); delirium at the time of assessment (n = 7); ineligible fracture type (n = 3); refusal to participate (n = 2); VAS ≤ 40 mm (n = 2); and BMI > 35 kg.m^‐2^ (n = 1). Sixty‐four patients were enrolled and randomly allocated: 32 to the PENG block group and 32 to the fascia iliaca block group. Two patients allocated the PENG block group did not receive the allocated intervention due to obesity‐related technical difficulty in performing the block in one case and an identified protocol violation (diaphyseal fracture) in another case, which should have precluded enrolment (Fig. 1). These two patients were considered dropouts and received standard emergency department care and analgesia. All patients were included in the intention‐to‐treat analysis, with the two patients in the PENG block group who did not receive the block considered as having a %SPID of zero. The 62 patients who received either intervention (PENG n = 30, fascia iliaca block n = 32) were included in the per protocol analysis. The two dropouts from the PENG group who did not receive the assigned intervention were not included in the analysis of the secondary outcome ‘rescue analgesia’. The two groups were well balanced in terms of baseline characteristics (Table 1).

**Table 1:** Baseline characteristics and pre‐enrolment analgesia of patients with hip fracture allocated to receive the pericapsular nerve group (PENG) block or fascia iliaca block. Values are median (IQR [range]) or number.

	PENG group, n = 32	Fascia iliaca block group, n = 32
Age; y	81 (75–86 [45–100])	82 (71–88 [49–93])
Sex; female	25	24
BMI; kg.m^‐2^	23.7 (20.2–26.1 [18.0–34.8])	24.2 (20.9–26.7 [18.4–33.6])
Pre‐block pain; mm VAS	90 (80–100 [60–100])	80 (70–91 [50–100])
Pre‐enrolment intravenous analgesia		
Paracetamol	32	32
NSAID	3	4
Opioid	4	4
Opioid dose; intravenous MME	3 (3–5 [3–10])	8 (6–10 [4–10])

MME, morphine milligram equivalents; NSAID, non‐steroidal anti‐inflammatory drug; VAS, visual analogue scale.

**Figure 2:**
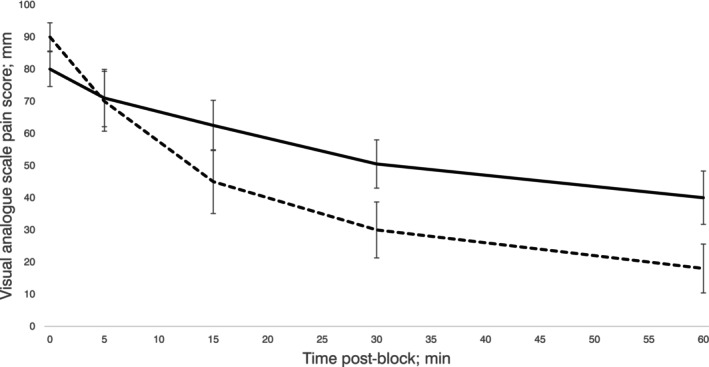
Median visual analogue scale pain scores shown over the 60‐min assessment interval for patients with hip fracture allocated to the pericapsular nerve group (PENG) block group (dashed line) and fascia iliaca block group (solid line). Data from participants who received their assigned interventions (per protocol analysis) are shown. Error bars represent 95%CI.

From the VAS data, the pain intensity differences and the primary outcome %SPID were calculated. For the primary outcome, patients allocated to the PENG block group showed a greater %SPID when compared with those allocated to the fascia iliaca block group (62.7% (95%CI 52.9–72.4%) vs. 38.0% (95%CI 30.7–45.4%), respectively; difference ‐24.7% (95%CI ‐36.6 to ‐12.7%), p < 0.001). Per protocol analysis showed similar results (online Supporting Information Figure S1). The clinically significant 33% SPID threshold was reached by 28/32 patients allocated to the PENG group and 19/32 patients allocated to the fascia iliaca block group (p = 0.022). In the PENG group, 24/32 patients achieved 50% SPID compared with 7/32 in the fascia iliaca block group (p < 0.001). Dynamic VAS pain scores (with 15° passive straight leg raise) over time for patients who received their assigned intervention are shown in Figure 2. More detailed dynamic pain score data from per protocol analysis are provided in online Supporting Information Table S1.

Rescue analgesia was administered to 5/30 patients allocated to the PENG group vs. 10/32 in those allocated to the fascia iliaca block group (p = 0.243), with a mean administered intravenous dose of 3.2 (95%CI 1.8–4.6) MME and 3.0 (95%CI 2.7–3.3) MME, respectively (p = 0.284). Three patients experienced opioid‐related adverse events: two (one in each group) had transient hypopnoea; and one patient allocated to the fascia iliaca block group reported dizziness. All events were self‐limited and resolved spontaneously without the need for any intervention. There were no other adverse events.

## Discussion

In this single‐centre randomised clinical trial conducted in an academic emergency department, the PENG block was superior to the infra‐inguinal fascia iliaca block in relieving acute pain in patients who presented with hip fractures.

The results of our study differ from a previous study by Marrone et al., in which the investigators found no difference between the same two regional block techniques [32]. There are some important differences between our study and that by Marrone et al. that may, at least in part, explain the divergent findings. First, the primary outcome in the study by Marrone et al. was the number of patients with post‐block pain scores ≤ 4 using a numeric rating scale, which does not consider change in pain scores from baseline or inter‐individual variation. Second, pain was measured only at a single time‐point 30 min after block placement, which may have been too soon to assess the full benefit of either block. Our study addresses these two methodological shortcomings by assessing pain over a period of 60 min post‐block and comparing groups based on %SPID. Third, Marrone et al. did not assess patients for suspected delirium or dementia with a formal assessment or validated tool; thus, potentially eligible patients could have been included or excluded inappropriately. Fourth, a higher dose of ropivacaine was used for the fascia iliaca block compared with the PENG block in the Marrone et al.'s study, while the same dose of local anaesthetic was used in both blocks in our study.

The superior analgesic effect of the PENG block may be due to its direct local anaesthetic spread around the anterior capsule, where it effectively blocks distal sensory fibres from the obturator, accessory obturator and femoral nerves, which are typically spared by the fascia iliaca block [33]. In addition, when the PENG block is performed with a minimum volume of 20 ml, the local anaesthetic solution can spread further distally to the intertrochanteric region [33], where it may act directly on the fracture site.

Our study shows an analgesic advantage favouring the PENG block over the infra‐inguinal fascia iliaca block, which is a recommended regional block approach for patients with hip fracture according to guidelines [4]. However, these results should not be generalised to all fascia iliaca block techniques. There is evidence suggesting that the supra‐inguinal approach for the fascia iliaca block may provide superior analgesia when compared with the infra‐inguinal approach [34, 35]. To date, comparisons of the PENG block with the supra‐inguinal fascia iliaca block in terms of hip analgesia have not yet established one to be clearly superior [12, 16, 19], and there may be a role to combine these two techniques since their respective targets may produce complementary analgesia [36]. In the peri‐operative period, the PENG block has been shown to produce less motor block than the supra‐inguinal fascia iliaca block with comparable analgesia, which is considered an advantage in terms of early mobility after surgery [37].

Our findings may have important implications, particularly in emergency medicine. While the fascia iliaca block is currently considered ‘best practice’ in emergency departments as part of multimodal pain management for patients with hip fractures [38], the results of our study, along with data from peri‐operative studies by anaesthetists supporting better analgesia and preservation of muscle function, suggest that this recommendation may need reconsideration in the future. More evidence is needed to confirm our findings, but for now, the PENG block should at least be considered an alternative to the fascia iliaca block, particularly in situations in which quadriceps motor block may be undesirable. There may be challenges in teaching the PENG block to emergency physicians. The fascia iliaca block, especially when using the infra‐inguinal approach, is generally viewed as easy to learn and safe due to the superficial location of the target plane, while the PENG block is deeper and may be viewed as riskier in patients who are taking anticoagulant or antiplatelet medications. There may be challenges in teaching the PENG block to emergency medicine trainees as even the consultant physicians involved in performing our study procedures were more experienced in the fascia iliaca block. However, there are signs of change, as regional anaesthesia courses tailored specifically for emergency physicians have started to include the PENG block in their programmes, as well as the management of complications [39, 40].

A major limitation of this study is its recruitment at a single centre. While originally planned with two sites of enrolment, the inability to set up the UK site in time made it necessary to revise the protocol. The setting of the study was the emergency department and all regional block procedures were performed by emergency medicine consultants. This system of practice may differ from other centres in which all blocks may be performed by anaesthetists regardless of location. Thus, the results of our study may not be widely generalisable and require further validation through similar research at other centres, or preferably a large multicentre study in the emergency department setting. Another limitation is the 1 h duration of post‐block pain score measurement, which was selected to specifically assess the potential analgesic benefits of each block post‐injury without interference from other clinical activities (e.g. transport, positioning changes for imaging or examination). This limited time interval did not account for the full duration of block effect or the total impact on pain, pain interference or opioid consumption while the block was in effect. Since the patients in our study had not yet undergone surgery, these results can only be applied to short‐term post‐injury acute pain and cannot be extrapolated to the postoperative period. Finally, data on patient chronic medical conditions, comorbidity burden and ASA physical status were not collected and therefore could not be analysed.

In summary, based on this single‐centre randomised clinical trial involving patients with hip fractures in an emergency department, the PENG block provides superior analgesia for the first hour after intervention when compared with the infra‐inguinal fascia iliaca block and represents a promising modality for acute pain management by physicians in this setting.

## Supporting information


**Appendix S1.** Regional anaesthesia in emergency medicine research group list of collaborators.

**Appendix S2.** Original study design.

**Figure S1.** Per protocol analysis for the primary outcome %SPID.

**Table S1.** Dynamic visual analogue scale pain scores in mm for the two treatment groups over the 1 h interval post‐block.

Plain Language Summary
