# Supplementary material for: Comparing the pericapsular nerve group block and fascia iliaca block for acute pain management in patients with hip fracture: a randomised clinical trial
Source: Anaesthesia. 2025 Jul 29;80(12):1484–92. doi: 10.1111/anae.16695 (PMC12614414; doi:10.1111/anae.16695)
Supplement: Supplementary file 1 — Appendix S1. Regional anaesthesia in emergency medicine research group list of collaborators. [file ANAE-80-1484-s004.docx]

**Online Supporting Information**

**Appendix S1.** **Regional Anaesthesia in Emergency Medicine (RAEM) research group list of collaborators.**

Federica Alini^1^

Valentina Angeli^1^

Bruno Barcella^1^

Rebecca Bersani^1^

Clara Bettini^1^

Marco Bonzano^1^

Vincenzo Capozza^1^

Letizia Caseti^2^

Alberto Castelli^3^

Annalisa Ceruti^1^

Pietro Costa^3^

Carlotta Cremaschi^1^

Fabrizio Cuzzocrea^3^

Salvatore D’Amico^3^

Emanuele Dal Fuoco^1^

Andrea Simone Dedato^1^

Guido Forini^3^

Federica Fossati^3^

Matteo Ghiara^3^

Elena Lago^1^

Irene Macaluso^1^

Luca Mangini^3^

Sara Mariucci^2^

Giuseppe Mignosa^1^

Greta Monne^1^

Mario Mosconi^3^

Cristina Naturale^1^

Fabrizio Paparella^3^

Nicola Parisi^1^

Gianluigi Pasta^3^

Claudio Pavesi^3^

Francesco Pelillo^3^

Michela Alessandra Pierro^1^

Michele Rendina^3^

Francesco Salinaro^1^

Annunziata Santaniello^1^

Valeria Sergi^1^

Antonino Tavella^3^

Damiano Vignaroli^1^

Jessica Zanovello^3^

1) Emergency Medicine Unit and Emergency Medicine Postgraduate Training Program, IRCCS Fondazione Policlinico San Matteo, Department of Internal Medicine, University of Pavia, Pavia, Italy

2) Biostatistics and Clinical Trial Center, IRCCS Fondazione Policlinico San Matteo, Pavia, Italy

3) Operative Unit of Orthopaedics and Traumatology, IRCCS Fondazione Policlinico San Matteo, Department of Clinical, Surgical, Diagnostic and Pediatric Sciences, University of Pavia, Pavia, Italy
