# Supplementary material for: Comparing the pericapsular nerve group block and fascia iliaca block for acute pain management in patients with hip fracture: a randomised clinical trial
Source: Anaesthesia. 2025 Jul 29;80(12):1484–92. doi: 10.1111/anae.16695 (PMC12614414; doi:10.1111/anae.16695)
Supplement: Supplementary file 2 — Appendix S2. Original study design. [file ANAE-80-1484-s005.pdf]

PENG!

BLOCK FOR HIP FRACTURE ANALGESIA

Pericapsular Nerve Group (PENG) Block versus Fascia Iliaca (FIB) Block for Emergency Department Analgesia in Hip Fractures: A Multicenter, Single-blind, Randomised Controlled Trial.

This trial will be registered at *clinicaltrial.gov* once obtained ethical approval.
The authors, investigators and participants do not receive any funding or incentives for the conduction of the study.

Name and affiliations of Investigators

Santi Di Pietro, MD, S.C. Pronto Soccorso Accettazione, Fondazione IRCCS Policlinico San Matteo, Pavia
PhD Student in Experimental Medicine, University of Pavia, Italy

Flavia Resta, MD, Emergency Medicine Postgraduate Training Program, IRCCS Policlinico San Matteo Foundation, Department of Internal Medicine, University of Pavia, Italy

Mattia Kolletzek, Senior Emergency Medicine Registrar, Colchester General Hospital, Colcheser, United Kingdom

Benedetta Mascia, Servizio Anestesia e Rianimazione 2, Fondazione IRCCS Policlinico San Matteo, University of Pavia, Pavia, Italy

Guido Forini, Department of Orthopaedics and Traumatology, Fondazione Policlinico IRCCS San Matteo, University of Pavia, Pavia, Italy.

Jegendirabose Parthipan, Consultant in Anaesthesia, East Suffolk and North Essex NHS Foundation Trust

Annalisa De Silvestri, Clinical Epidemiology and Biometry Unit, Fondazione IRCCS Policlinico San Matteo, Pavia, Italy.

Valeria Musella, Biometry and Clinical Epidemiology Service, Fondazione IRCCS Policlinico San Matteo, Pavia, Italy.

Stefano Perlini, Direttore S.C. Pronto Soccorso Accettazione, Fondazione IRCCS Policlinico San Matteo, Pavia, Emergency Medicine Fellowship Program, University of Pavia

Name and affiliations of Sub-investigators

Bruno Barcella, MD, Emergency Medicine Postgraduate Training Program, IRCCS Policlinico San Matteo Foundation, University of Pavia, Italy

Cristina Naturale, MD, Emergency Medicine Postgraduate Training Program, IRCCS Policlinico San Matteo Foundation, University of Pavia, Italy

Nunzia Santaniello, MD, Emergency Medicine Postgraduate Training Program, IRCCS Policlinico San Matteo Foundation, University of Pavia, Italy

Marco Bonzano, MD, S.C. Pronto Soccorso Accettazione, Fondazione IRCCS Policlinico San Matteo, Pavia

Gianmarco Secco, MD, S.C. Pronto Soccorso Accettazione, Fondazione IRCCS Policlinico San Matteo, Pavia

Damiano Vignaroli, MD, S.C. Pronto Soccorso Accettazione, Fondazione IRCCS Policlinico San Matteo, Pavia

Greta Monne, Medical Student, University of Pavia

Emilio Carfì, Medical Student, University of Pavia

Roles and Responsibilities

Dr Santi Di Pietro is the principal investigator (PI) for the IRCCS Policlinico San Matteo Foundation. The PI coordinates all phases of the study, from the development of the study protocol to the enrolment of participants, conduction of the experimentation, data analysis and dissemination of the study results. The PI for Colchester Emergency Department will be Mattia Kolletzek. The PI will also be responsible for communication with the ethical board and will guarantee the safekeeping of participants' data.

'Investigators' are consultant physicians and trainees from various specialties who have accepted to participate in the study (Emergency Medicine, Anaesthesiology, Orthopaedics) and working in IRCCS Policlinico San Matteo Foundation (Pavia, Italy) and Colchester General Hospital (Colchester, UK).
They will provide scientific support in their respective area of expertise and, together with the PIs and CRC, they contribute to the study development at all stages, from drafting of the protocol to the dissemination of results.

Sub-investigators are Emergency Medicine trainees from the Emergency Medicine Postgraduate Training Program of the University of Pavia (Director Prof. Stefano Perlini) and medical students from the same University. Under the supervision of the PI, they provide support in literature search, writing of the study protocol, recruitment of volunteers, results analysis and dissemination of results.

Introduction

Background and rationale

Hip fractures are regarded as a worldwide epidemic and a major public health concern in many countries, representing a significant cause of morbidity and mortality worldwide.
Globally, during the year 2000, there were an estimated 1.6 million hip fractures accounting for about 20% of all fractures in people aged 50 years and older [[1]-[2]]. Pain management is a crucial aspect of the care of hip fracture patients presenting to Emergency Departments (EDs).

[1] Johnell O, Kanis JA. An estimate of the worldwide prevalence, mortality and disability associated with hip fracture. Osteoporos Int. 2004 Nov;15(11):897-902. doi: 10.1007/s00198-004-1627-0. Epub 2004 May 4. PMID: 15490120.

[2] Rapp, K., Büchele, G., Dreinhöfer, K. *et al.* Epidemiology of hip fractures. *Z Gerontol Geriat* **52,** 10–16 (2019). https://doi.org/10.1007/s00391-018-1382-z

Untreated or poorly treated pain increases the risk of delirium [3-4], which in turn is closely related to mortality [5]. In addition to that, perioperative analgesia has a direct impact on the hospital length of stay, time to first mobilisation, risk of chest infection and long-term functional impairment [6-7]. Current research highlights the importance of an early-delivered, high-quality, multimodal analgesia in hip fractures, ideally minimising the opioid consumption to reduce opioid-related side effects [8-9-10]. For this purpose, several scientific societies endorse the use of regional anaesthesia techniques, in particular fascia iliaca block

---

[3] Daoust R, Paquet J, Boucher V, Pelletier M, Gouin É, Émond M. Relationship Between Pain, Opioid Treatment, and Delirium in Older Emergency Department Patients. Acad Emerg Med. 2020 Aug;27(8):708-716. doi: 10.1111/acem.14033. Epub 2020 Jun 11. PMID: 32441414.

[4] Lynch EP, Lazor MA, Gellis JE, Orav J, Goldman L, Marcantonio ER. The impact of postoperative pain on the development of postoperative delirium. Anesth Analg. 1998 Apr;86(4):781-5. doi: 10.1097/00000539-199804000-00019. PMID: 9539601.

[5] Aung Thein, M.Z., Pereira, J.V., Nitchingham, A. *et al.* A call to action for delirium research: Meta-analysis and regression of delirium associated mortality. *BMC Geriatr* **20,** 325 (2020). https://doi.org/10.1186/s12877-020-01723-4

[6] Morrison SR, Magaziner J, McLaughlin MA, Orosz G, Silberzweig SB, Koval KJ, Siu AL. The impact of post-operative pain on outcomes following hip fracture. Pain. 2003 Jun;103(3):303-311. doi: 10.1016/S0304-3959(02)00458-X. PMID: 12791436.

[7] Guay J, Kopp S. Peripheral nerve blocks for hip fractures in adults. Cochrane Database Syst Rev. 2020 Nov 25;11(11):CD001159. doi: 10.1002/14651858.CD001159.pub3. PMID: 33238043; PMCID: PMC8130997.

[8] Benyamin R, Trescot AM, Datta S, Buenaventura R, Adlaka R, Sehgal N, Glaser SE, Vallejo R. Opioid complications and side effects. Pain Physician. 2008 Mar;11(2 Suppl):S105-20. PMID: 18443635.

[9] Sanzone AG. Current Challenges in Pain Management in Hip Fracture Patients. J Orthop Trauma. 2016 May;30 Suppl 1:S1-5. doi: 10.1097/BOT.0000000000000562. PMID: 27101319.

[10] Fabi DW. Multimodal Analgesia in the Hip Fracture Patient. J Orthop Trauma. 2016 May;30 Suppl 1:S6-S11. doi: 10.1097/BOT.0000000000000561. PMID: 27101321.

(FIB) and femoral nerve block, for the management of pain in patients diagnosed with hip fractures at emergency departments [11-12]. Performing fascia iliaca block has now become a standard procedure for emergency physicians across the globe.

A Cochrane review on these techniques used in hip fracture patients demonstrated an average pain score reduction of 2.5 on a 10-point Numerical Rating Scale, 30 minutes after the nerve blocks [13]. Although this analgesic effect exceeds the minimal clinically important pain reduction, recent international guidelines have questioned the routine use of these blocks in the context of patients with a fractured neck of femur [14-15].

An anatomical study by Short et al [16] provided an explanation for the modest analgesic effect of traditional blocks (FIB and FN block) for neck of femur fractures. In fact, they demonstrated that sensory innervation of the anterior capsule of the hip is provided by articular branches of the femoral, obturator and accessory obturator nerve. Almost all

---

[11] Royal College of Emergency Medicine, Best Practice Guideline, Fascia Iliaca Block in the Emergency Department 2020

[12] American College of Emergency Physicians, Policy Statement, Ultrasound-guided nerve blocks, April 2021

[13] Guay J, Kopp S. Peripheral nerve blocks for hip fractures in adults. Cochrane Database Syst Rev. 2020 Nov 25;11(11):CD001159. doi: 10.1002/14651858.CD001159.pub3. PMID: 33238043; PMCID: PMC8130997.

[14] Myles PS, Myles DB, Galagher W, et al. Measuring acute postoperative pain using the visual analog scale: the minimal clinically important difference and patient acceptable symptom state. Br J Anaesth 2017;118:424–9.

[15] National Clinical Guideline Centre (NCGC) at the Royal College of Physicians. Chapter 7: Analgesia, paragraph 7.3. In: The management of hip fracture in adults. London, UK, 2019. https://www.nice.org.uk/guidance/cg124/evidence/full-guideline-pdf183081997

[16] Short AJ, Barnett JJG, Gofeld M, et al. Anatomic study of innervation of the anterior hip capsule: implication for image-guided intervention. Reg Anesth Pain Med 2018;43:186–92.

cadavers examined (92%) had "high" sensory articular branches that originate cranially to the inguinal ligament; therefore, these nerve terminations are unlikely to be blocked with the traditional infra-inguinal techniques.

In addition to that, fascia iliaca block rarely results in obturator nerve block [[17]].

Based on these findings, Giron-Arango et al developed in 2018 the pericapsular nerve group block (PENG) for hip fracture. This block consists in an ultrasound-guided injection of local anaesthetic in the musculofascial plane between the psoas tendon and the ileopubic eminence, resulting in a pericapsular spread of the anaesthetic agent. In their original paper, the authors described a median 7-points pain reduction on a 10-point scale following PENG block in patients with hip fractures, with similar findings reported by other researchers [[18]-[19] -[20]]. These preliminary findings have generated enthusiasm about the use of this block, which according to many clinical practitioners provides superior analgesia as compared to the standard approaches, although this belief is currently supported by weak evidence. To date, only one recent randomised double-blind study has compared the analgesic efficacy of PENG

[17] Weller RS. Does fascia iliaca block result in obturator block? Reg Anesth Pain Med. 2009 Sep-Oct;34(5):524; author reply 524. doi: 10.1097/AAP.0b013e3181ada59f. PMID: 19749590.

[18] Morrison C, Brown B, Lin D-Y, et al. Reg Anesth Pain Med Epub ahead of print: [please include Day Month Year]. doi:10.1136/rapm-2020- 101826

[19] Del Buono R, Padua E, Pascarella G, Costa F, Tognù A, Terranova G, Greco F, Fajardo Perez M, Barbara E. Pericapsular nerve group block: an overview. Minerva Anestesiol. 2021 Apr;87(4):458-466. doi: 10.23736/S0375-9393.20.14798-9. Epub 2021 Jan 12. PMID: 33432791.

[20] Sahoo RK, Jadon A, Sharma SK, Nair AS. Pericapsular nerve group (PENG) block for hip fractures: Another weapon in the armamentarium of anesthesiologists. J Anaesthesiol Clin Pharmacol. 2021;37(2):295-296. doi:10.4103/joacp.JOACP_295_20

block versus suprainguinal FIB [21]. The authors demonstrated a superior analgesia using PENG block to allow patient positioning for spinal anaesthesia prior to hip surgery [21]. To the best of our knowledge no trials have compared PENG block with the standard approach in hip fracture patients in the setting of Emergency Departments.

Objectives

The main objective of this study is to compare the pain relief experienced by hip-fracture patients in the first hour after receiving PENG block or FIB. Secondary objectives are the frequency of satisfactory pain relief and the opioid requirement in the first hour following either block, as well as the safety profile of the two approaches based on the adverse events experienced during ED stay.

Study design

We designed a multicentric, randomised-controlled trial with two parallel study groups. Participants will be randomised 1:1 to the study or control group. The study group will be treated with PENG block following randomisation, whereas the control group will receive the standard treatment, i.e., fascia iliaca block. The study aims to demonstrate the superiority of the new intervention over the standard approach.

---

[21] Jadon, Ashok; Mohsin, Khalid; Sahoo, Rajendra K1; Chakraborty, Swastika; Sinha, Neelam; Bakshi, Apoorva Comparison of supra-inguinal fascia iliaca versus pericapsular nerve block for ease of positioning during spinal anaesthesia, Indian Journal of Anaesthesia: August 2021 - Volume 65 - Issue 8 - p 572-578
doi: 10.4103/ija.ija_417_21

Methods: Participants, interventions, and outcomes

Study setting

The trial will be conducted at the Emergency Department *S.C. Pronto Soccorso Accettazione* (Director Prof. Stefano Perlini) *of the IRCCS Fondazione Policlinico San Matteo* (Pavia, Italy) and at the Emergency Department of Colchester General Hospital (Colchester, United Kingdom).
Both departments have an annual caseload of > 300 hip fractures. This caseload should guarantee the completion of the study within the time described later in this protocol.

*Due to difficulties in setting up the second recruiting centre in the UK, on May 20th 2023 we submitted an amendment to the ethics committee requesting to continue recruitment only in Pavia as a single-centre study.

Eligibility criteria

We will propose participation to the study to patients who meet the following criteria:

- Age >18
- Capacity to understand the aim of the study, the potential benefits and side-effects of the procedures
- Capacity to provide consent
- Capacity to provide a self-assessment of pain using the written VAS Scale
- Confirmed radiological diagnosis of hip fractures (including subcapitate,

transcervical, intertrochanteric and perthrocanteric fractures)

- Moderate or severe worst pain (visual analogue scale, VAS >40 mm) (at rest or dynamic)

Exclusion criteria:

- Known hypersensitivity to local anaesthetics
- Confirmed radiological diagnosis of subtrochanteric or diaphyseal femur fractures
- Hemodynamic instability
- Known diagnosis of severe cognitive impairment
- Dementia and/or delirium (defined by a 4AT score ≥ 2)
- Lack of capacity to provide consent and to understand the aim of the study
- BMI > 35
- Body weight < 40 Kg
- Prior hip surgery on the same fracture side
- Mild worst pain (visual analogue scale, VAS < 40 mm) (at rest or dynamic)

Cognitive status will be evaluated using the 4AT test (see Supplementary material – File A). This is a screening test for a rapid and sensitive initial assessment of cognitive impairment and delirium. It explores alertness, attention, change of fluctuation in mental function and space-time orientation. As described by Bellelli et al. in their original paper, a score of 4 or higher suggests delirium [[22]]. More recently, O'Sullivan et al. validated the 4AT for the diagnosis of

[22] Bellelli G, Morandi A, Davis DH, et al. Validation of the 4AT, a new instrument for rapid delirium screening: a study in 234 hospitalised older people [published correction appears in Age Ageing. 2015 Jan;44(1):175]. Age Ageing. 2014;43(4):496-502. doi:10.1093/ageing/afu021

delirium as well as dementia in older Emergency Department attendees [23]. The authors found that a cut-off of 1/2 (i.e., 0 or 1 as normal) effectively rules out delirium (sensitivity 0.74; specificity 0.87; PPV 0.61; NPV 0.92). In addition to that, they also developed a solution to overcome the need for family or carer for collateral information, which is needed for the assessment of Item 4. When a collateral was missing, they adopted the following algorithm: if items 1–3 were normal and the patient had no hallucinations, delusions or fluctuations during assessment, or reported by ED staff, then item 4 was scored '0'; if items 2–3 were abnormal (total score 1–3), then item 4 was scored '4' (to avoid missed diagnosis). The sensitivity and specificity for delirium detection was unaffected by this algorithm [2].

Based on these findings, we will use the 4AT with a 1/2 cut-off to screen for dementia and delirium. Whenever a collateral history will be missing, we will apply the algorithm described above.

Body mass index (BMI) will be calculated using the standard formula (BMI = kg/m2) where kg is a person's weight in kilograms and m2 is their height in square metres.

Interventions

All eligible patients who meet the inclusion and exclusion criteria will be invited to participate in the study by one of the investigators listed in this protocol. After having signed the informed consent, participants will be randomised 1:1 to either receive the FIB or PENG block. Participants will be instructed to report their pain level using the visual analogue scale (VAS),

---

[23] O'Sullivan D, Brady N, Manning E, et al. Validation of the 6-Item Cognitive Impairment Test and the 4AT test for combined delirium and dementia screening in older Emergency Department attendees. Age Ageing. 2018;47(1):61-68. doi:10.1093/ageing/afx149

that consists of a 100 mm line, from 0 ("no pain") to 100 ("pain as bad as it could possibly be"). They will be given a little folder consisting of 5 sheets on which the VAS scale is printed. Each sheet corresponds to a measurement of pain level that will take place before the block is performed (T0), and 5-15-30-60 minutes following the block (T1-T2-T3-T4). Participants will rate their pain level by placing a mark on each of the five VAS scales.
Pain will be assessed by the treating physician both at rest and upon gentle passive straight leg raise of the affected limb to 15° (dynamic). Patients will be asked to report the worst pain experienced during the assessments.

FIB and PENG block will be performed by a small group of physicians (4 ED consultants in Pavia, 4 senior registrars/consultants in Colchester) with advanced ultrasound skills. These physicians have local certifications in advanced ultrasound competencies and are actively involved in ultrasound education, teaching and research. They all routinely use US-guided fascia iliaca block in their clinical practice. Prior to the beginning of the study, they will receive an ad-hoc training on PENG block (see further "*Participating medical personnel*").

The equipment needed for the procedures, including ad-hoc needles for regional anaesthesia, local anaesthetics, probe covers, sterile gloves and skin preparation solution (Clorexidine 2%) will be stored in a dedicated trolley in each ED. The trolley will also contain lipid emulsion (Intralipid 20%) to be used in case of local anaesthetic toxicity. All blocks will be performed using standard sterile precautions and under continuous monitoring (ECG, NIBP, pulse oximeter). Standard safety measures will be adopted, including "STOP before your block"

checklist [24] and aspiration prior to injection.

All nerve blocks will be performed under sonographic guidance. The ultrasound machine that will be used at both centres is a SonoSite Edgell..

During the 60 minutes following administration of a block, further intravenous analgesia can be administered if needed or requested by the patient. However, physicians will be asked to only prescribe iv morphine (0.05 mg/Kg) during this time frame, which can be repeated after 30 minutes if needed. After 60 minutes from the block, i.e., following the last per-protocol pain assessment, patients will receive a multimodal analgesia as according to local practice and with no restrictions in terms of drug choice (clinical pathways if present, or alternatively physicians' preferences).

Study group

Patients enrolled in the study group will receive a PENG block with 30 mL of 0,25% levobupivacaine with 4 mg of dexamethasone. The block will be performed with the patient in a supine position using an 18-gauge, 90 mm needle, inserted with an in-plane lateral to medial approach. Operators will use the original technique described by Girón-Arango L et al [25]. The

---

24 https://www.ra-uk.org/images/stories/documents/CSQ-PS-sbyb-posterA4.pdf

25 Girón-Arango L, Peng PWH, Chin KJ, Brull R, Perlas A. Pericapsular Nerve Group (PENG) Block for Hip Fracture. Reg Anesth Pain Med. 2018 Nov;43(8):859-863. doi: 10.1097/AAP.0000000000000847. PMID: 30063657.

aim of this block is to inject the local anaesthetic between the psoas tendon and the iliopubic eminence.

We will instruct operators to routinely use a curvilinear probe (2-6 MHz). Nevertheless, they will have the option to use a linear probe (4-16 MHz) in particularly lean or cachectic patients.

Control group

Patients allocated in the control group will receive an infrainguinal fascia iliaca block with 30 mL of 0,25 % levobupivacaine with 4 mg of dexamethasone. The block will be performed with the patient in supine position using an 18-gauge, 90 mm needle, inserted with an in-plane lateral to medial approach.

For ultrasound guidance a linear (4-16 MHz) probe will be used.

The probe is placed transversely at the inguinal crease to identify the femoral artery, femoral nerve, iliopsoas muscle and the fascia iliaca over the psoas muscle. Moving the probe laterally the sartorius muscle and the anterior inferior iliac spine (AIIS) can be identified. After skin disinfection the needle is inserted placing the tip beneath the fascia iliaca at the lateral third of a line between the AIIS and pubic tubercle. Correct needle placement is confirmed by separation of the fascia iliaca from the iliopsoas muscle upon injection, with local anaesthetic spreading towards the FN medially and the iliac crest laterally.

Participating medical personnel

All medical and nursing staff working in the participating institutions will be informed about the aim of the trial and will be instructed to identify potentially eligible patients and liaise with the researchers (investigators and sub-investigators listed above). Researchers will verify that patients meet the criteria to be included in the study, including the assessment of cognitive status using the 4AT. In addition to that, researchers will verify the presence on the shopfloor of at least one of the physicians with the prerogative to perform the procedures. Researchers will propose participation in the study and will ask patients to sign informed consent. Moreover, researchers will be responsible to assess the pain level at established intervals with the modalities described above and will instruct patients to report pain on the VAS scale.

The physicians with the prerogative to perform procedures are local experts in ultrasound and routinely perform FIB in their practice. Before the beginning of the study, they will attend a training session on PENG block. Training sessions will take place at both centres under the supervision of the CRC in cooperation with two senior anaesthesiologists (BM at IRCCS San Matteo and JP at Colchester Hospital – See List of Investigators) with extensive experience in regional anaesthesia. Teaching will include a theoretical part as well as practical hands-on scanning. The content of the training will be standardised and delivered in the same way in both institutions.

Outcome Measures

Primary outcome:

- Pain relief over the 60-minutes following either block measured as the percentage of summed pain-intensity difference (%SPID) (derived from VAS measurement at T0-T1-T2-T3-T4 as described above)

Secondary outcomes:

- Number of patients who achieve a percentage of summed pain-intensity difference of 33% (33%SPID)
- Number of patients who achieve a percentage of summed pain-intensity difference of 50% (50%SPID)
- Quantity of opioids (milligrams of morphine) administered in the first 60 minutes following either block
- Occurrence of adverse events including nausea or vomiting, hypotension, respiratory depression (hypoxia or hypopnea), local anaesthetic toxicity syndrome (LAST syndrome) during the ED stay (post-block)

SPID is a widely used variable to determine treatment response to analgesics over a period of time. This value is calculated using the pain-intensity difference (PID) at each time point. The PID is calculated as the change from baseline VAS for each measurement in time. SPID is the summation of PID at each of the study time points and weighted using the amount of time since the prior assessment; it approximates the area under the curve for PID over time.
The advantage of using SPID is that it considers individual differences in baseline pain intensity over time [[26]].

[26] Beaudoin FL, Haran JP, Liebmann O. A comparison of ultrasound-guided three-in-one femoral nerve block versus parenteral opioids alone for analgesia in emergency department

SPID can also be reported as a percentage of maximum possible SPID (%SPID). Maximum possible SPID is the value that would be achieved if the patient were pain free (VAS =0) for the entire study period. We are interested in the number of patients who achieve a %SPID of 33%, as this has been previously established to represent a clinically important measurement in pain outcomes [[27]].
Moreover, we are also interested in the number of patients who achieve a %SPID of 50%.
For what concerns safety, we aim to measure the occurrence of adverse events related to nerve blocks and to the use of opioids. Nausea or vomiting is defined as patient-reported nausea, documented emesis or administration of antiemetic drugs during ED stay (post-enrolment). Hypotension is defined as a systolic blood pressure below 100 mmHg at any time during ED stay (post-enrolment). Hypopnea is defined as a respiratory rate below 10 breaths/min. The occurrence of a LAST syndrome will be based on the clinical judgement of the treating physician. Two independent physicians (MK, FR) will retrospectively review patients' ED record to assess for other adverse events including naloxone administration, agitation or confusion, use of lipid emulsion. Although the study period for the purpose of pain evaluation is limited to one hour following the block, we will consider the overall length of stay in ED (post-block) for the occurrence of adverse events.

---

patients with hip fractures: a randomized controlled trial. Acad Emerg Med. 2013 Jun;20(6):584-91. doi: 10.1111/acem.12154. PMID: 23758305.

[27] Farrar JT, Berlin JA, Strom BL. Clinically important changes in acute pain outcome measures: a validation study. J Pain Symptom Manage. 2003 May;25(5):406-11. doi: 10.1016/s0885-3924(03)00162-3. PMID: 12727037.

Participant timeline

Following approval of the relevant ethical boards, we will recruit a prospective, non-consecutive sample of adults in our Emergency Departments.

Recruitment will take place on a 24/7 basis with no time restrictions. However, as previously described, recruitment will be subject to the presence on the shopfloor of at least one physician with the prerogative to perform the procedures.

Patients who are eligible for participation in the study will receive a full detailed explanation of the aim of the study. Moreover, they will be thoroughly informed about potential risks, side-effects and benefits of the interventions. Patients who accept to participate will sign one consent form for study participation, and a second form related to the procedures. The latter consent is the one routinely adopted in the clinical practice at both institutions (Modulo 14.1 at IRCCS Fondazione Policlinico San Matteo).

Once enrolled in the study, participants will be randomised using an online-based system (REDCap - Research Electronic Data Capture) to the intervention (PENG block) or control group (FIB block). After receiving either block, pain will be assessed with the modalities and intervals described above. Once completed the last pain assessment, i.e., 60 minutes following the block, patients will receive usual ED care as according to established local pathways/physician's preferences and will be hospitalised in orthopaedic wards.

We expect patient enrolment to last approximately 4-6 months since the approval of ethical boards.

Sample size

The sample size is calculated based on the available literature. Beaudoin et al. described a median (range) %SPID of 36.9 (-25 to 100) following “3-in-1” femoral nerve block [[28]], from which we derived a Standard Deviation of 33 using the formula described by Wan et al [[29]]. The “3-in-1” block has been demonstrated to be equivalent to fascia iliaca block in terms of analgesic efficacy, therefore we expect to observe similar percentages of %SPID [[30]]. Current studies on PENG block report an average pain reduction as high as 7 points on a 0 to 10 points scale [18-19-20]. If we consider a %SPID of 65 with PENG block a sample size of 29 subjects in each arm would provide 90% power to detect a difference between the two interventions, with a significance level of alpha < 0.05, enrolment ratio 1:1 and a common SD equal to 30. We have decided to inflate the sample size by 10% to account for attrition, missing data and protocol violations, resulting in a total of 32 subjects in each arm. Recruitment will be non-competitive, i.e. each centre will be expected to enrol half of the cases.

Methods: assignment of interventions

Allocation – Sequence generation

[28] Beaudoin FL, Haran JP, Liebmann O. A comparison of ultrasound-guided three-in-one femoral nerve block versus parenteral opioids alone for analgesia in emergency department patients with hip fractures: a randomized controlled trial. Acad Emerg Med. 2013 Jun;20(6):584-91. doi: 10.1111/acem.12154. PMID: 23758305.

[29] Wan, X., Wang, W., Liu, J. *et al.* Estimating the sample mean and standard deviation from the sample size, median, range and/or interquartile range. *BMC Med Res Methodol* **14,** 135 (2014). https://doi.org/10.1186/1471-2288-14-135

[30] Reavley P, Montgomery AA, Smith JE, Binks S, Edwards J, Elder G, Benger J. Randomised trial of the fascia iliaca block versus the '3-in-1' block for femoral neck fractures in the emergency department. Emerg Med J. 2015 Sep;32(9):685-9. doi: 10.1136/emermed-2013-203407. Epub 2014 Nov 27. PMID: 25430915.

Every participant will be identified with a number from one to fifty based on the chronological order of enrolment. The investigators will use an online-available platform for randomisation (REDCap - Research Electronic Data Capture). A 1:1 random block randomization list will be prepared stratified by center before the study initiation by an expert statistician at Clinical Epidemiology and Biometry Unit using the ralloc procedure implemented in STATA v 17.0-.

Blinding

Patients will not know what treatment they will receive. In addition to that, when performing the blocks physicians will keep the ultrasound screen away from patient sight, to prevent participants with ultrasound or anatomy skills from distinguishing the two approaches. In any case, even for a patient with medical/ultrasound knowledge it would be very difficult to recognize any difference between the two approaches, as both methods share almost identical sites of skin disinfection, scanning and needle insertion.
The statistician analysing study data will also be blinded. It is not feasible to blind the operator performing the procedures.

Methods: data collection, management, and analysis

Data collection instruments

Source documents

Study data are collected on source documents. The PIs are responsible for assuring that collected data are complete and accurate. Source documents include all recordings of

observations or notations of clinical activities and all reports and records necessary for the evaluation and reconstruction of the clinical study.

Data collection and study report form monitoring

All data obtained for this study will be entered into a local regulation compliant Data Management System [for reference ex: 21 CFR Part 11 (USA)]. This is provided by the Service of Biometry & Clinical Trial Center (CTC) of the I.R.C.C.S. Policlinico San Matteo Foundation (Pavia, Italy). Data will be recorded with an Electronic Data Capture (EDC) system using eCRFs. Specifically, the EDC will be based on the RedCap platform. REDCap is a novel workflow methodology and software tool that expedites the electronic collection of research data from a single site or multi-site clinical research study. The software supports a secure web-based application for developing fully functional case report forms (CRFs) and surveys. In particular, through RedCap we will implement: (a) Full user authentication (log-on/password) to restrict users to study functions; (b) Real-time data validation, integrity checks for ensuring data quality; (c) De-identification options to be applied to data exports to remove fields that contain notes and other information that could identify patient; (d) Centralized, secure storage of research data with back-ups; (e) The study database will be resident on a server in a secure location within the I.R.C.C.S. Policlinico San Matteo Foundation, Pavia, Italy.

The CI will ensure the accuracy and completeness of the data reported to the DMC. All data entry, modification or deletion will be recorded automatically in an electronic audit trail. The CI will retain all copies of the eCRF in the relevant sections of their Investigator Site File with any required anonymised background information from the medical records as required.

Data management

All patients' files (consents, folders for pain measurement, CRFs) will be stored in a safe site located within the Emergency Departments of the two institutions for data monitoring and analysis. The PIs are responsible for transferring all paper-collected data to the electronic CRF.

Statistical methods

A full data management plan and a full statistical analysis plan (SAP) will be drafted and approved by the scientific board prior to study start. After SAP signature by the scientific board (i.e., prior to any statistical analysis), the study database will be locked. Any changes to the protocol-specified or SAP-specified planned analyses that are made after the database lock will be described in the clinical study report.

Statistical analyses

Descriptive statistics will be obtained for all variables assessed in the study population. Mean and standard deviation will be used for normally distributed variables, mean and interquartile range for skewed distributions, proportions for categorical variables. Whenever relevant, 95% confidence intervals (95%CI) will be calculated. P-value <0.05 will be considered significant. In all cases, two-tailed tests will be applied.
Stata v 17.0 software will be used for statistical analysis.

Primary endpoint

Treatment groups will be compared by means of parametric t-test for independent samples or Mann-Whitney nonparametric test for quantitative variables (according to distribution; normality will be tested by means of the Shapiro–Wilk test).

Secondary endpoints

- Number of patients who achieve a percentage of summed pain-intensity difference of 33% (33%SPID)

  Treatment groups will be compared by means of Pearson's $\chi2$ test (Fisher exact test where appropriate) for categorical variables.
- Number of patients who achieve a percentage of summed pain-intensity difference of 50% (50%SPID)

  Treatment groups will be compared by means of Pearson's $\chi2$ test (Fisher exact test where appropriate) for categorical variables
- Quantity of opioids (morphine equivalents) administered in the first 60 minutes following either block

  Treatment groups will be compared by means of parametric t-test for independent samples or Mann-Whitney nonparametric test for quantitative variables (according to distribution; normality will be tested by means of the Shapiro–Wilk test).
- Occurrence of adverse events including nausea or vomiting, hypotension, respiratory depression (hypoxia or hypopnea), local anaesthetic toxicity syndrome (LAST syndrome) during ED stay (post-block). Adverse event will be described, and their incidence will be presented with 95% CI and compared with Fisher test.

Safety and adverse events

We aim to measure the occurrence of adverse events related to nerve blocks and to the use of opioids. In particular we will report the following adverse events: nausea or vomiting, hypotension, hypopnea and the occurrence of a LAST syndrome. A detailed definition of adverse events is provided in the "Secondary outcome" section. Although the study period for the purpose of pain evaluation is limited to one hour following the block, we will consider the overall length of stay in ED (post-block) for the occurrence of adverse events. All adverse events will be reported in the CRF.

Ethics and dissemination

This protocol will be submitted for ethical approval to the relevant ethical committees ("Comitato Etico Pavia"; "East Suffolk - North Essex NHS Ethics Board"). All participants will sign an informed consent that includes an explicit request to authorise the dissemination of anonymised study results. In addition to that, patients will also be asked to sign a separate consent form to allow the physicians perform the procedures (Modulo per l'acquisizione del consenso informato alle procedure diagnostiche e/o terapeutiche - 14.1 - for the IRCCS Policlinico San Matteo).

Dissemination policy

Patient's data will not be shared with third parties and will always be used respecting their privacy.

At the end of the study, the investigators will produce a scientific paper for submission to peer-reviewed journals in the field of emergency medicine. In case of publication, all investigators and sub-investigators will be mentioned as co-authors.
The authors of the publications will be decided on the basis of indications contained in the Uniform Requirements for Manuscripts (http://www.icmje.org/urm_full.pdf).

Funding

This study is not sponsored.
Conduction of the study does not add additional costs for the participating institutions, as the anaesthetic drugs, the needles, the syringes and the ultrasound are routinely available and used in the EDs involved.
