# Supplementary figures and images for: Comparing the pericapsular nerve group block and fascia iliaca block for acute pain management in patients with hip fracture: a randomised clinical trial
Source: Anaesthesia. 2025 Jul 29;80(12):1484–92. doi: 10.1111/anae.16695 (PMC12614414; doi:10.1111/anae.16695)

**Figure S1.** Per protocol analysis for the primary outcome %SPID.


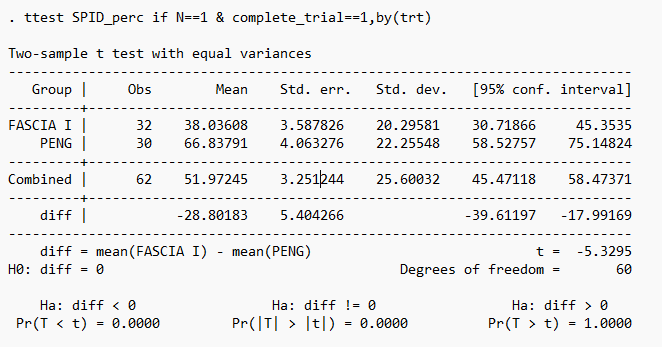

Supplement: Supplementary file 3 — Figure S1. Per protocol analysis for the primary outcome %SPID. [file ANAE-80-1484-s003.docx]
