# Supplementary material for: Comparing the pericapsular nerve group block and fascia iliaca block for acute pain management in patients with hip fracture: a randomised clinical trial
Source: Anaesthesia. 2025 Jul 29;80(12):1484–92. doi: 10.1111/anae.16695 (PMC12614414; doi:10.1111/anae.16695)
Supplement: Supplementary file 4 — Table S1. Dynamic visual analogue scale pain scores in mm for the two treatment groups over the 1 h interval post‐block. [file ANAE-80-1484-s001.docx]

**Table S1.** Dynamic visual analogue scale pain scores in mm for the two treatment groups over the 1 h interval post-block. Values are median (IQR [range]).

|  | **PENG (n = 30)** | **FIB (n = 32)** |
| --- | --- | --- |
| **T_0_ (Baseline)** | 90 (80–100 [60–100]) | 80 (70–91.2 [50–100]) |
| **T_1_ (5 minutes)** | 70 (55–82.5 [0–100]) | 71 (57.5–90 [0–100]) |
| **T_2_ (15 minutes)** | 45 (23–65 [0–90]) | 62.5 (48.7–80 [0–100]) |
| **T_3_ (30 minutes)** | 30 (12.5–40 [0–74]) | 50.5 (40–66.2 [0–90]) |
| **T_4_ (60 minutes)** | 18 (0–27.5 [0–70]) | 40 (30–52.2 [0–90]) |

FIB, fascia iliaca block; PENG, pericapsular nerve group
