# Supplementary material for: Comparing the pericapsular nerve group block and fascia iliaca block for acute pain management in patients with hip fracture: a randomised clinical trial
Source: Anaesthesia. 2025 Jul 29;80(12):1484–92. doi: 10.1111/anae.16695 (PMC12614414; doi:10.1111/anae.16695)
Supplement: Supplementary file 5 — Plain Language Summary [file ANAE-80-1484-s002.docx]

**Plain Language Summary**

When people break their hip, they usually feel a lot of pain. Doctors use special kinds of pain relief to help them feel better. This includes nerve blocks to make the hip feel numb. One type is called the fascia iliaca block, and another newer one is the PENG block. This study was done in a hospital emergency department to see which block worked better at reducing pain quickly for people with a hip fracture. Doctors randomly chose which block each patient would get. They picked patients who had a broken hip and were in moderate or worse pain. One group got the PENG block, using 20 ml of a numbing medicine with a small amount of another helpful drug. The other group got the fascia iliaca block, using a little more of a slightly weaker numbing medicine and the same extra drug. The main goal was to see how much the pain went down after the block. Other things they checked were: how many patients had at least a third or half less pain; how much extra pain medicine (like morphine) was needed; and if anyone had bad side effects. Out of 92 patients checked, 64 joined the study (32 in each group). Patients who got the PENG block had a bigger drop in pain than those who got the fascia iliaca block. In the PENG block group, 28 out of 32 patients felt at least a third less pain, compared to 19 out of 32 in the other group. Both groups needed about the same amount of extra pain medicine. The PENG block helped reduce pain better in the first hour after treatment than the fascia iliaca block. It looks like a good option for helping patients with hip fractures feel better in the emergency department.
